# A farm-level study of risk factors associated with the colonization of broiler flocks with *Campylobacter *spp. in Iceland, 2001 – 2004

**DOI:** 10.1186/1751-0147-49-18

**Published:** 2007-07-10

**Authors:** Michele T Guerin, Wayne Martin, Jarle Reiersen, Olaf Berke, Scott A McEwen, John-Robert Bisaillon, Ruff Lowman

**Affiliations:** 1 Department of Population Medicine, Ontario Veterinary College, University of Guelph, Guelph, Ontario, N1G 2W1, Canada; 2 Reykjagarður hf, Fosshals 1, 112 Reykjavík, Iceland; 3 Agricultural Agency of Iceland, Austurvegur 64, 800 Selfoss, Iceland; 4 Department of Biometry, Epidemiology and Information Processing, University of Veterinary Medicine Hannover, Bünteweg 2, D-30559 Hannover, Germany; 5 Canadian Food Inspection Agency, Ottawa, Ontario, K2H 8P9, Canada

## Abstract

**Background:**

Following increased rates of human campylobacteriosis in the late 1990's, and their apparent association with increased consumption of fresh chicken meat, a longitudinal study was conducted in Iceland to identify the means to decrease the frequency of broiler flock colonization with *Campylobacter*. Our objective in this study was to identify risk factors for flock colonization acting at the broiler farm level.

**Methods:**

Between May 2001 and September 2004, pooled caecal samples were obtained from 1,425 flocks at slaughter and cultured for *Campylobacter*. Due to the strong seasonal variation in flock prevalence, analyses were restricted to a subset of 792 flocks raised during the four summer seasons. Flock results were collapsed to the farm level, such that the number of positive flocks and the total number of flocks raised were summed for each farm. Logistic regression models were fitted to the data using automated and manual selection methods. Variables of interest included manure management, water source and treatment, other poultry/livestock on farm, and farm size and management.

**Results:**

The 792 flocks raised during the summer seasons originated from 83 houses on 33 farms, and of these, 217 (27.4%) tested positive. The median number of flocks per farm was 14, and the median number of positive flocks per farm was three. Three farms did not have any positive flocks. In general, factors associated with an increased risk of *Campylobacter *were increasing median flock size on the farm (p ≤ 0.001), spreading manure on the farm (p = 0.004 to 0.035), and increasing the number of broiler houses on the farm (p = 0.008 to 0.038). Protective factors included the use of official (municipal) (p = 0.004 to 0.051) or official treated (p = 0.006 to 0.032) water compared to the use of non-official untreated water, storing manure on the farm (p = 0.025 to 0.029), and the presence of other domestic livestock on the farm (p = 0.004 to 0.028).

**Conclusion:**

Limiting the average flock size, and limiting the number of houses built on new farms, are interventions that require investigation. Water may play a role in the transmission of *Campylobacter*, therefore the use of official water, and potentially, treating non-official water may reduce the risk of colonization. Manure management practices deserve further attention.

## Background

*Campylobacter *spp. remain one of the most frequent bacterial causes of foodborne gastroenteritis around the world [1]. Poultry, and specifically consumption of undercooked poultry and mishandling raw poultry, is thought to be an important source of *Campylobacter *to humans [2-7]. The prevalence of broiler flocks colonized with *Campylobacter *spp. varies among countries, ranging from 5% of flocks to more than 90% [8]. Once a flock is exposed, the bacteria spread rapidly through the flock, and most of the birds become colonized and remain so until slaughter [9-14]. In Iceland, the incidence of domestically-acquired human campylobacteriosis peaked in 1999 at 117.6 cases per 100,000 persons [15], and sampling of broiler carcasses and domestic human cases from August to October 1999 showed that 85% of *Campylobacter *isolates in humans had identical genetic sequences (*flaA *SVR) to isolates from broilers [16]. Due to the difficulties in eliminating contamination of carcasses in slaughter plants, the control of *Campylobacter *in broiler flocks and subsequent production of birds free from colonization at slaughter is essential for preventing human cases [5,14,17-19].

Several epidemiological studies have examined risk factors for the colonization of broiler flocks with *Campylobacter*. Farm-level factors associated with an increased risk of colonization include: the presence of other animals on the farm [13,20-23]; the presence of other poultry nearby [12]; manure disposal inside the farm [23]; greater than 200 m between the broiler house and the nearest manure heap (versus ≤200 m) [24]; farm water supply [25]; providing broilers with non-disinfected drinking water [26]; increasing number of birds raised per year on the farm (which was highly correlated with the number of broiler houses on the farm) [24] and increasing flock size [12]. These factors were identified using univariable and multivariable statistical methods to examine a large number of risk factors that potentially act at the flock, house or farm level. To our knowledge, there are no farm-level studies that have attempted to delineate risk factors that specifically influence the proportion of positive flocks on a farm.

The strong association between the increased incidence of human campylobacteriosis and increased consumption of fresh chicken meat in Iceland, prompted a longitudinal study of the poultry industry [27]. The ultimate goal of the full project was to identify the means to decrease the frequency of broiler flock colonization with *Campylobacter*, thereby reducing the burden of foodborne illness associated with poultry consumption. Our objective in this study was to identify risk factors for flock colonization acting at the broiler farm level.

## Methods

### Target and study populations

The target population for our study was commercial broiler flocks raised in Iceland between May 2001 and September 2004. Our initial plan was a total-population census sampling of all flocks from commercial broiler production farms in a three-year longitudinal study beginning in May 2001; sampling was later extended by five months to include a fourth peak summer season. Sampling was carried out in the three commercial abattoirs in the South of Iceland, where commercial production was located. This level of full cooperation by broiler producers was in part due to public and media attention to the link between broiler chickens and the campylobacteriosis epidemic in 1999, and due to a price penalty to producers for positive flocks. Producers and processors were keenly interested in finding solutions. When the study began in May 2001, only one farm was excluded, due to its remote location and small production. The excluded farm slaughtered its own small flocks in an on-farm facility and sold its products locally. It ceased production in April 2003 and as best we can determine by hatchery records, it raised 12 flocks during the study period with a maximum flock size of 3,000 birds and total production of less than 36,000 broilers. During the course of the study several new farms joined the study with their first flocks. Two of these new farms were excluded due to their remote location. One was located on a coastal island, distant from the study area. It raised only two flocks with a total of 14,900 broilers before closing operations. The other excluded farm was located on the North coast of Iceland, with production exceeding initial expectations. It produced 147 flocks with a total of 1,241,026 broilers during the course of the study and an average flock size of 8,442 birds. Including the estimated production from the first excluded farm, only 161 flocks (contributing 11% of the total broiler production in Iceland during the study period) were excluded from the study. Of the 1,425 flocks included in the study (total production of 10,387,169 broilers), the maximum flock size was 23,470 birds, the mean flock size was 7,289, and the median flock size was 6,142 birds. There were 47 flocks with less than 2,000 broilers, and 36 flocks with over 20,000 broilers.

### Characteristics of the farms

Commercial broiler flock production technology in Iceland is essentially the same as that in North America and Europe. Breeder production of hatching eggs, hatchery technology, broiler ventilation, feeding and water delivery systems are the same. Scale of production is smaller on average, with newer broiler barns being state of the art and more comparable in size to large scale production elsewhere. Icelandic broiler houses have concrete floors and floor drainage systems as the standard, which is a notable difference from broiler barns in North America, especially the US. A number of farms also use geothermal water to heat the broiler houses and to wash out the pens. Farms can be a mixture of newer, larger broiler houses, with original houses being smaller, whereas newer farms will have only newer-style, large broiler houses.

### Data collection

Pilot sampling of broiler flocks, including pooled caecal samples, were conducted for three months of production in 2000. General data on the characteristics of each farm were gathered at the beginning of the study through a combination of phone interviews and site visits by the Veterinary Officer for Poultry Diseases of the Agricultural Agency of Iceland (Reiersen). Collection of epidemiological data began in May 2001. Since no problems were encountered during the initial collection of data, all sampling data were included in the dataset for analysis. Questionnaire designs were the collective effort of five veterinarians (including four epidemiologists) and a biostatistician. Included in the design group was the Veterinary Officer for Poultry Diseases, who had an in-depth knowledge of each farm as a result of working with the producers to eradicate *Salmonella *from poultry. There were several questionnaires, the main one designed to record independent variables acting at the various levels of broiler production (i.e. at the flock, house and farm levels). During the interval between flocks in each broiler house, a field technician employed by the Veterinary Officer for Poultry Diseases visited each farm to record responses from face-to-face interviews with the person most closely associated with the hands-on management of the broiler flocks and houses, and to record observations of cleaning and disinfection procedures between flocks. The design team reviewed all questions and the method of recording with the field technician to ensure clear understanding. The Veterinary Officer for Poultry Diseases accompanied the field technician on all farm visits and questionnaire recording for the first full month of sampling. During the course of the study, two university-educated field technicians were employed. The first technician was employed for two years, and trained the second technician for one month prior to leaving the project. Interview times varied from 10 to 15 minutes per questionnaire, depending on whether the producer needed to verify records. To ensure consistency in responses, data collected at the previous visit were reviewed with the producer. All questions pertaining to our analysis were closed. Although other factors potentially relevant to the complex epidemiology of *Campylobacter *were included in the questionnaires, it was our intent in this study to specifically identify risk factors operating at the farm level. The set of factors chosen for this analysis were deemed both sensible and comprehensive to satisfy the objectives of this study and were in keeping with farm-level factors identified in the literature.

The slaughter plants provided additional data, in the form of monthly reports summarizing records of flocks slaughtered each day.

### Bacteriological sampling and processing

Depending on the size of the flock and management practices on the farm, broiler flocks were shipped to the slaughter plant in one to four catch lots, defined as a group of birds collected on one day and transported to the slaughter plant. The maximum trucking distance was 100 km. In Iceland, live haul crates and trucks are cleaned and disinfected with great care, and there are no commercial catching crews (i.e. all flocks are caught by each farm's workers). Caecal samples were chosen to ensure representation of farm-origin flock *Campylobacter *status, and for their higher sensitivity compared to cloacal swabs or faecal samples. At the processing plants, systematically selected caeca (including contents) were excised from 40 birds from each catch lot by veterinarians employed by the Chief Veterinary Office of Iceland and placed in sterile WhirlPac bags to create four pooled samples containing ten caeca each. The caeca were collected from the viscera pack of carcasses on the evisceration line in the abattoir, after automatic evisceration. Flock slaughter lots are well-separated in Icelandic abattoirs, which facilitates clear flock identification. The sampling protocol was to select an indicator carcass (not sampled), and then collect one caecum from each 10th or 5th subsequent carcass, whichever frequency worked best for work flow. Caeca were collected using one pair of latex examination gloves per pooled sample; gloves were changed between pooled samples. Caeca were removed by manually freeing an individual caecal loop from connective tissue, pinching it off at its base, and pulling it free. Samples were then transported and processed at the Laboratory of the Institute for Experimental Pathology, Keldur, Iceland, either the same day or after holding overnight at 4°C. The required sample size per flock was estimated to detect early stages of flock colonization or alleles with poor colonizing ability on the basis of a within-flock prevalence as low as 10%; four pooled samples would ensure 99% confidence of detecting at least one positive bird in a catch lot [28]. Serial dilutions of caecal contents were plated on Campy-Cefex agar [29] and incubated at 42°C under microaerobic conditions for 48 hours. Colonies were counted, and confirmed as *Campylobacter *spp. by microscopy and latex agglutination (DrySpot *Campylobacter *test kit, Oxoid DR0150M).

Although enumeration was not required for this epidemiological analysis, the choice of a direct plating method that enabled enumeration was important to other aspects of the large multi-disciplinary project. Campy-Cefex was chosen due to low cost, good sensitivity and enumeration on caecal samples. The method requires 24 to 48 hours for confirmed detection of *Campylobacter *spp. (versus at least 72 hours for the NMKL method), enabling identification of positive flock lots to obtain retail product samples prior to distribution (two cartons of ready-to-ship broiler carcasses were held pending caecal sample results for another component of the full project). During the first eight months of sampling, caecal samples were analysed by both methods [30]. Based on the results of this comparison, the Laboratory of the Institute for Experimental Pathology concluded that the Campy-Cefex method was at least as sensitive as the NMKL method for detecting *Campylobacter *spp. in poultry caecal samples, and began using Campy-Cefex for their official Icelandic surveillance program. Since genetic sub-typing was deemed necessary for other project analyses, we were unable to go further into the species identification of the isolates.

### Outcome

A broiler flock was considered positive for *Campylobacter *if at least one of the pooled samples from any of the catch lots was positive on culture. Data were then collapsed to the farm level, such that the number of positive flocks and the total number of flocks raised were summed for each farm.

### Summer data

Since the clear majority of positive flocks in our study were detected during the warmer months, we focussed our analysis on flocks raised during this high risk period to reduce problems associated with interactions of management factors with season [31]. Thus, flocks that hatched between March 15 and September 15 of each year of the study were considered to have been raised during the summer season. This definition of summer corresponds to the periods of restrictions imposed by the Icelandic government on when manure is allowed to be spread on fields and pasture (March 15 to October 31).

### Definition of farm-level variable

A farm-level variable was defined as one that was consistent for all houses on a farm during the study period. However, as can be expected over a three and a half year study, producers may have instituted changes at the farm level such that flocks raised in the early part of the study were subjected to a different management practice or circumstance than flocks raised in the latter part. In this situation, if at least 80% of the flocks from a farm were subjected to a particular management practice, then that practice was deemed to be the standard for the farm.

### Variables

Table 1 lists the categorical variables that were available for analysis. Only farms with complete data for all variables (28 farms) are shown since only these farms were included in the multivariable analysis described below. Due to the small number of farms, for categorical predictors with more than two levels, categories were combined if it was biologically sensible to do so. Continuous predictors are summarized in Table 2.

**Table 1:** Farm-level categorical variables available for analysis of *Campylobacter *colonization of broilers in Iceland (n = 28^a^)

Variable	Description of variable	Number of farms
Manure spread on fields in summer season^b^	Yes	11
	No	17
Manure spread on fields in winter season^b^	Yes	9
	No	19
Manure stored on farm at any time of year	Yes	10
	No	18
Other domestic livestock on farm	Yes	9
	No	19
Other commercial poultry on farm	Yes	10
	No	18
Farm has all-in-all-out policy	Yes	18
	No	10
Farm water source	Non-official	17
	Non-official treated	4
	Official	4
	Official treated	3

^a ^Only farms with complete data for all variables are included

^b ^Variables are highly correlated (τ_b _≥ 0.8)

**Table 2:** Farm-level continuous variables available for analysis of *Campylobacter *colonization of broilers in Iceland (n = 28^a^)

Variable	Minimum	50% percentile	Maximum
Number of houses on farm	1.0	2.0	15.0
Median flock size on farm	2667.5	6142.5	19109.5

^a ^Only farms with complete data for all variables are included

Initial screening of categorical variables consisted of identifying those that were highly correlated with each other (Kendall's τ_b _≥ 0.8) (Table 1).

### Multivariable modelling

Since the goal of our model-fitting process was primarily aimed at identifying the most important of the farm-level predictors, we examined a number of potentially useful models. Six logistic regression models, using the binomial distribution to adjust for the number of flocks from each farm, were fitted to the data using both automated and manual variable selection methods. The models were of the following form:

ln [p_*i*_/(1-p_*i*_)] = β_0 _+ β_1_X_1*i *_+ ... + β_k_X_k*i*_

where p_*i *_is the proportion of positive flocks from farm *i*, β_0 _is the intercept, and β_1_X_1*i *_+ ... + β_k_X_k*i *_is the linear combination of predictor variables for the *i*^th ^farm.

Four different automated model selection methods were applied to fit adequate models using any of the predictor variables listed in Tables 1 and 2. When manual selection methods were employed, only one of the two strongly correlated variables for manure spreading was included with the remaining available predictors. The model selection procedures were as follows: 1) automated forward selection; 2) automated forward stepwise; 3) automated backward selection; 4) automated backward stepwise; 5) manual backward selection using the variable "manure spread on fields in summer season"; and 6) manual backward selection using the variable "manure spread on fields in winter season". For all models, the test of a term's significance was the likelihood-ratio test; variables with p < 0.05 were eligible for addition to the model and p ≥ 0.05 were eligible for removal. The likelihood-ratio test was also used to evaluate the significance of groups of variables (i.e. farm water source). Akaike's Information Criterion (AIC) was recorded for each model. The linear relationship between each continuous predictor and the outcome was evaluated by adding a quadratic term to the regression model and assessing its significance, with p ≤ 0.05 indicating a non-linear relationship. In the manual selection methods, as each variable was removed from the model, confounding was deemed to exist, and the variable was retained in the model, if the coefficient of another significant variable changed by more than 20%. With one exception (see discussion), interactions were not assessed since there were relatively few farms. Model diagnostics included the calculation of Pearson residuals to identify outliers; observations with large residuals were further evaluated by re-fitting the model without the observation and comparing the coefficients to the full model. Potential influential observations were identified by examining large Cook's distance values. Stata software version 9 (StataCorp, College Station, TX, USA) was used for all statistical analyses.

## Results

### Descriptive summary

Data were available for 792 flocks on 33 farms, and of these, 217 (27.4%) tested positive for *Campylobacter*. The median number of flocks per farm was 14 (mean 24, range 1 to 146), and the median number of positive flocks per farm was 3 (mean 7, range 0 to 55). The proportion of positive flocks per farm ranged from 0 to 75%, with a median and mean of 25%. Three farms did not have any positive flocks; these were primarily smaller farms that each raised a total of 1 to 9 flocks during the four summer seasons of the study. Other domestic livestock on farms included cattle only (1 farm), pigs only (1 farm), sheep only (2 farms), and sheep plus cattle and/or horses (5 farms).

Of the 792 flocks raised during the four summer seasons, the total production was 5,828,772 broilers. This figure was slightly less (5,659,534 broilers) for the 28 farms (758 flocks) included in the multivariable analyses. The median age at slaughter of flocks raised during the summer seasons was 37 days (mean 37, range 31 to 100). The age distribution for flocks included in the multivariable analyses was similar although the maximum age was 63 days. The number of houses per farm ranged from 1 to 15 (median 2, mean 2.5). Individual flocks ranged in size from 604 to 21,772 broilers (median 6,275, mean 7,366). A large proportion of flocks (72%) were slaughtered in one catch lot. For flocks with more than one catch lot, the mean catch lot size was 5,065 broilers (range 330 to 14,867). Each catch lot was sampled at slaughter. Of the 217 positive flocks, 14 flocks were slaughtered in three catch lots with four samples per catch lot for a total of 12 samples per flock, 46 flocks were slaughtered in two catch lots with four samples per catch lot for a total of eight samples per flock, and the remaining 157 flocks were slaughtered in one catch lot with four samples per flock. On the basis of catch lot sampling, out of 291 catch lots, 266 were positive in all samples (91.4%), 2 were positive in three samples (0.7%), 6 were positive in two samples (2.1%), and 17 were positive in one sample (5.8%). On a flock basis, 14 of the 217 positive flocks were positive in only one pooled sample, likely indicating early stages of flock colonization.

The characteristics of farms excluded from the analyses due to missing data for one or more variables are shown in Table 3. There were no obvious patterns among the excluded farms other than none of the farms raised other livestock and all had either one or two houses. The proportion of positive flocks on these farms ranged from 25% to 75%.

**Table 3:** Characteristics of farms excluded from the farm-level analyses due to missing data

Variable	Farm A	Farm B	Farm C	Farm D	Farm E
Manure spread on fields in summer season	No	---^a^	---	---	---
Manure spread on fields in winter season	No	---	---	---	---
Manure stored on farm at any time of year	No	---	---	---	---
Other domestic livestock on farm	No	No	No	No	No
Other commercial poultry on farm	Yes	No	Yes	No	No
Farm has all-in-all-out policy	---	Yes	No	Yes	Yes
Farm water source	Official	Official	Official treated	Non-official treated	Non-official
Number of houses on farm	1	1	2	2	1
Median flock size on farm	4019	1323	7971	4190	6728
Number of flocks raised	4	4	8	14	4
Number of positive flocks	3	1	2	5	1

^a^Data not available

### Multivariable analysis

The variables "manure spread on fields in summer season" and "manure spread on fields in winter season" were strongly and positively correlated with each other (τ_b _= 0.86). Of the 28 farms included in the multivariable analysis, there were 9 farms that spread manure in both summer and winter, 17 farms that did not spread manure in either season, and 2 farms that spread manure in the summer but not in the winter.

Coefficients and p-values for the variables in each model are presented in Table 4. For categorical variables, exponentiation of the coefficient represents the increase (positive coefficient) or decrease (negative coefficient) in the risk of *Campylobacter *when the factor was present on the farm compared to when it was not present on the farm. For example, using the coefficient of 0.92 from the manual backward selection model using "manure spread in summer", the risk of a flock being colonized with *Campylobacter *was 2.5 times higher (e.g. e^0.92 ^= 2.5) on farms that spread manure on fields in the summer season compared to farms that did not spread manure in the summer. Exponentiation of the coefficient for the continuous variables represents the increase in the risk of *Campylobacter *as the median flock size increased by 1,000 birds, and the increase in risk for each additional house on the farm (see discussion). The p-values in Table 4 represent the probability that the increase or decrease in risk was due to chance alone. For example, the p-value of 0.025 for manure spreading in the summer indicates that there was a 2.5% probability that the observed increased risk of *Campylobacter *colonization was due to chance.

**Table 4:** Six logistic models for farm-level factors associated with *Campylobacter *in broilers in Iceland (n = 28^a^)

Variable	Automated forward selection	Automated forward stepwise	Automated backward selection	Automated backward stepwise	Manual backward selection using "manure spread in summer"	Manual backward selection using "manure spread in winter"
Median flock size on farm (divided by 1,000)	0.10 (0.000)^b^	0.10 (0.000)	0.11 (0.000)	0.09 (0.001)	0.13 (0.000)	0.12 (0.000)
Farm water source						
Non-official			Ref.	Ref.	Ref.	Ref.
Non-official treated			-0.57 (0.066)	-0.03 (0.913)	-0.50 (0.169)	-0.33 (0.370)
Official			-0.60 (0.051)	-0.76 (0.009)	-1.04 (0.004)	-0.99 (0.005)
Official treated			-0.87 (0.032)	-1.06 (0.006)	-1.21 (0.006)	-1.17 (0.007)
Other domestic livestock on farm			-0.65 (0.026)	-0.60 (0.028)	-1.01 (0.004)	-0.90 (0.004)
Other commercial poultry on farm	0.59 (0.000)	0.59 (0.000)			-0.72 (0.063)	-0.59 (0.097)
Number of houses on farm			0.06 (0.038)		0.13 (0.008)	0.11 (0.008)
Manure spread on fields in winter season^c^			0.54 (0.035)	1.90 (0.014)	Not included in model	0.97 (0.004)
Manure spread on fields in summer season^c^				-1.71 (0.028)	0.92 (0.025)	Not included in model
Manure stored on farm at any time of year					-0.83 (0.029)	-0.76 (0.025)
Farm has all-in-all-out policy						
AIC^d ^of model	5.09	5.09	4.86	4.78	4.94	4.79

^a ^Only farms with complete data for all variables are included

^b ^Regression coefficient (p-value).

^c ^Variables are highly correlated (τ_b _≥ 0.8)

^d ^Akaike's Information Criterion

For each variable in Table 4, a range of coefficients and p-values are presented. The values differ depending on the model selection method. The presence of data in the table is an indication that the variable was associated with *Campylobacter *colonization in the respective model, whereas the absence of data indicates that the variable was not associated with flock colonization (i.e. the variable was either removed (backward-type models) or it was not eligible for addition (forward-type methods)). The variables are listed in descending order, such that factors identified as being associated with *Campylobacter *in all models are the top of the table. For example, increasing median flock size was identified as a strong risk factor in all six models, whereas an all-in-all-out policy was not a significant predictor in any of the models. Factors that were significantly associated with colonization regardless of modelling approach could be considered to have a greater relative importance in the epidemiology of *Campylobacter *on broiler farms in Iceland.

In general, the factors associated with an increased risk of *Campylobacter *were increasing median flock size, spreading manure on the farm in the winter, and increasing the number of broiler houses on the farm. Protective factors included the use of official or official treated water on the farm compared to the use of non-official untreated water, storing manure on the farm at any time of year, and the presence of other domestic livestock on the farm.

In the automated forward selection and forward stepwise models, one farm had a large residual (standardized Pearson's residual = 3.2) relative to the residuals of the other farms. The characteristics of this farm were: non-official water, one house, an all-in-all-out system, manure was spread and stored at all times of the year, absence of other livestock and poultry, and a mean flock size of 4,579 birds. Although this farm had a much higher proportion (7/15) of positive flocks than predicted (2.5/15), it did not have undue influence on the models.

## Discussion

A high relative frequency of a variable being included in the various models, and a consistent association with flock colonization across models (Table 4), may help indicate the true causal role of that factor, and hence the potential for producers to decrease risk on the farm by applying an appropriate intervention directed at that factor. Median flock size, followed by farm water source and the presence of other domestic livestock on the farm, were the factors that were included in most or all of the models. Spreading manure on the farm in the winter season was present in 60% of the models, the number of broiler houses was present in 50%, and storing manure on the farm at any time of year was present in one-third of the models. An all-in-all-out policy at the farm level (i.e. the practice of shipping all flocks on the farm within the span of a few days, with all houses remaining empty for a period of time) was not a significant predictor in any of the models. The direction of association was inconsistent for the presence of other commercial poultry on the farm and spreading manure on the farm in the summer season, and the statistical significance of the former was also inconsistent among models. In general, the models that employed a backward elimination approach had slightly smaller AIC's and a larger number of significant predictors than those using a forward approach. Backward selection has an advantage over forward and stepwise selection procedures in that negatively confounded sets of predictors are less likely to be omitted from the model [32]. Thus, more emphasis could be placed on variables identified as significant in the backward-type models.

An increased risk of *Campylobacter *was associated with increasing median flock size on the farms. For example, as the average flock size increased by 5,000 birds, the risk of *Campylobacter *colonization increased by approximately 57% to 92% (i.e. 1.57 to 1.92 times). Our findings are in contrast with several studies [22-24,26] that utilized multivariable logistic regression at the flock level, in which an association between flock size and *Campylobacter *status was not found. In a one-year study of 18 Swedish broiler farms, infection risk increased when the flock size was more than 25,000 birds [12]. However, the authors noted that since only univariable associations were examined, their conclusions may have been confounded by farm size and management practices. To our knowledge, ours is the first study to examine the effect of the average flock size on the farm on the risk of *Campylobacter *colonization. It has been suggested that larger flocks require more water, feed, litter, air and personnel, all possible sources of the bacteria [12]. Thus, in our study, increasing median flock size may be a surrogate for many other factors.

In our study, an official (municipal) water source was one in which the water was tested regularly for coliform bacteria by the municipality and treated if necessary, and an official treated water source was one in which the water was treated consistently with either ultraviolet (UV) light or a heat-cool method at the municipal level. We found that farms using official water sources had approximately one-third to half the risk of *Campylobacter *than farms using non-official untreated sources (the referent group). Similarly, farms using official treated water sources had roughly one-third the risk. These findings suggest that some flocks may have been exposed through contaminated water, as water has been identified as a suitable reservoir and medium for *Campylobacter *spp. [33]. Several studies [23,34-36] have found that there was no association between the occurrence of *Campylobacter *in flocks supplied with municipal (public) water compared to those supplied with well (private) water, however, in those studies, there was no distinction between the use of treated and untreated water sources. We found that farms using a non-official UV-treated water supply did not have a significantly different risk of *Campylobacter *than farms using non-official untreated water at the 5% level of significance, although in one model, non-official treated water did have a protective effect at a 10% level. Some researchers [25,26] have found that water disinfection had a protective effect on the colonization of broilers with *Campylobacter*, although others [24,36,37] have not found such an association. The small number of farms using non-official treated water in our study, combined with potential confounding by other factors, may account for the wide range in p-values for this variable. Our results suggest that the use of municipal water (both treated and untreated) reduces the risk of *Campylobacter *colonization of broiler flocks, and that some potential also exists for decreasing risk through the practice of treating non-official water sources, depending in part on other management practices on the farm. It is possible that there may be other, more indirect factors contributing to the risk of colonization, such as animal density in the region. In addition, there may be complex relationships between access of livestock to the water source, type of water source (drilled versus upcoming wells), and the method of water treatment (UV versus heat-cool) that were not adequately addressed in this study. Dissection of these inter-relationships would require a study in a country or region with a larger number of farms.

The presence of other domestic livestock on the farm was associated with a decreased risk of *Campylobacter *colonization. Similar results were obtained when we assessed the effect of the presence of cattle, rather than the presence of other domestic livestock in general. These findings were unexpected and inconsistent with other studies as it has been suggested that other domestic livestock species (especially cattle) may act as reservoirs that potentially contaminate the farm environment thereby providing a continual source of bacteria to the birds [13]. Several studies have shown that the presence of other animals on the farm (pigs, cattle, sheep, or fowl other than broilers) [20], (cattle) [21], (pigs, cattle, sheep and goats, or horses) [22], (laying hens, sheep, cattle, donkeys) [23] was associated with an increased risk of *Campylobacter*, although one recent Canadian study did not find such an association (cattle, sheep, goats, horses and/or pigs) [24]. However, in one Norwegian study [26], the presence of other poultry or animals at the farm was not associated with increased colonization of flocks, rather, tending other poultry and tending pigs prior to entering the broiler house were independently associated with an increased risk. In our study, farms that did and did not keep other domestic livestock were similar with respect to the number of flocks raised and the number of houses, both surrogates of farm size. The distance between the broiler houses and the housing for the other livestock is quite variable among broiler farms in Iceland, with distances ranging from immediately adjacent to approximately 900 m apart. Additionally, consistent patterns among farms in the management of other species (e.g. manure management, assignment of workers dedicated to a specific species, etc.) were not observed during farm visits, although specific questions on such management practices were not included in our questionnaires. Our findings may reflect that Icelandic producers that raise domestic livestock in addition to broilers take precautions that prevent contamination of the broiler houses, such as increased efforts at biosecurity and sanitation practices.

An increased risk of *Campylobacter *was associated with increasing numbers of broiler houses on the farms. For each additional house on the farm, the risk of *Campylobacter *colonization increased by approximately 6% to 14%. Although we analysed this factor as a continuous variable, our finding is consistent with several other studies [12,22,24,36]. There was a positive correlation (τ_b _= 0.75, p < 0.001) between the number of houses on the farm and the number of flocks raised on the farm. To determine if the increased risk was indeed associated with increasing numbers of houses, rather than just increasing numbers of flocks, we included the number of flocks as an independent predictor in the models and found that the number of broiler houses remained statistically significant, while the number of flocks was not significant. Several houses on the same farm may lead to an increased risk of *Campylobacter *through the introduction of the bacteria into the house from the environment [36], possibly through the increased movement of farm workers between houses, or difficulty in maintaining strict hygiene or biosecurity practices. In general, broiler farm workers in Iceland are not specific to a house. However, on farms that have both breeder and broiler houses, workers are generally assigned to either the broiler or breeder houses and producers take precautions with any exceptions. Each broiler house has its own set of boots and clothing, and in most cases, there is a strict separation and physical barrier between the exterior personnel entry area (for removal of outside boots and clothing), and the inside clean area with dedicated broiler house boots, coveralls, and hand wash and disinfectant. However, given the increased risk associated with increasing numbers of houses, for new broiler farms, consideration should be given to limiting the number of houses built.

The practice of storing manure on the farm was associated with a decreased risk of colonization and was an unexpected finding. We considered that this protective effect may be a result of producers storing manure when there was not enough space to spread it in the immediate vicinity, however, a brief exploration of the interaction between manure storing and spreading showed that the two factors were independent (regardless of modelling approach). One possible theory for our finding is that manure stored in large piles (as is the practice in Iceland) may be subjected to a form of composting or fermentation, which may be detrimental to the survival of the organism. By contrast, spreading manure on fields in the winter season was associated with an increased risk of *Campylobacter *colonization, although it is unclear how this practice increases risk. The effect of spreading manure on fields in the summer season varied depending on the model. In the automated backward stepwise model, multicollinearity was a problem, thus, the protective effect may be a spurious result because of its strong positive association with spreading manure in the winter. There is very little information about these predictors in the literature, and it is uncertain whether these practices are unique to Iceland. In Senegalese broiler flocks, an elevated risk of *Campylobacter *colonization was associated with manure disposal inside the farm compared to disposal outside the farm, presumably through continual contamination of the environment [23], although the nature of disposal was not stated. Similar to our findings, in Québec, Canada, the presence of a manure heap ≤200 m from the broiler house (versus > 200 m) was associated with a decreased risk of colonization, although the authors considered that this unexpected finding was the result of confounding by farm size [24]. Analysis of these risk factors in future studies, and studies that evaluate the survival of *Campylobacter *in manure under various environmental conditions, may substantially improve our understanding of the relationship between the farm environment and *Campylobacter *in broiler flocks.

A limitation of automated variable selection procedures is the potential for inclusion of strongly correlated variables in the model. In the automated backward stepwise model, the predictors "manure spread on fields in summer season" and "manure spread on fields in winter season" were both retained. The standard errors for these variables were slightly inflated (0.8 in this model compared to approximately 0.3 in other models) as a result of multicollinearity, therefore, the coefficients must not be over-interpreted. Notwithstanding this, these risk factors were significant in other models suggesting their importance in predicting the risk of *Campylobacter *on broiler farms in Iceland.

A second limitation of automated variable selection procedures is the inability to identify and evaluate potential confounding variables. In both automated forward models, the presence of other commercial poultry on the farm was associated with an increased risk of *Campylobacter *colonization. This finding is in agreement with one study [12], although other researchers [26,34,36] have not found an association. However, in both manual selection models, the presence of other commercial poultry was shown to be a confounder for most of the other predictors (including number of houses, farm water source, manure spreading and storing practices, and the presence of other livestock), and this accounts for the discrepancy between models. Sampling of sexually immature and parent breeder flocks in Iceland between May and July 2000, showed that up to 72% of faecal samples were positive for *Campylobacter *spp. [15], suggesting the potential for contamination of the farm environment from these other poultry. Our results show that after controlling for other farm-level factors, keeping other commercial poultry on the farm is not associated with the colonization of broiler flocks with *Campylobacter *in Iceland. However, in our classification of other poultry, we did not differentiate between those farms that raised turkey flocks and broiler flocks alternatively in the same house (with full cleaning and disinfection between flocks), from those farms that also have year round permanent breeder or egg layer flocks. With few exceptions, the latter tend to be constantly heavily contaminated *Campylobacter *reservoirs (based on sampling results of the on-going Icelandic surveillance program). Future studies should carefully classify other poultry on the farm in order to fully assess their impact on the risk of colonization of broiler flocks.

The variable "farm has an all-in-all-out policy" changed on one farm during the study. Since less than 80% of the flocks on this farm were subjected to this management practice, we deemed that the farm did not use an all-in-all-out system. In order to assess what effect this might have had on our models, we re-analysed the data using a repeated measures approach and found that the results were not affected. The repeated measures approach allowed the predictor to vary for different flocks raised on the same farm (i.e. flocks raised in the early part of the study were subjected to an all-in-all-out system, whereas, those in the latter part were not), and adjusted the standard errors to account for intragroup correlation. Regardless of statistical approach, this variable was one of the first to be removed in all of the backward elimination procedures and was not eligible for addition in any of the forward selection methods. Thus, in the Icelandic broiler industry, an all-in-all-out policy on the farm does not appear to be associated with *Campylobacter *colonization during the summer season. One possible explanation for this finding may be related to the changes in the broiler industry that took place following the epidemic in 1999 and the implicated role of fresh broiler chicken products. Broiler producers came under much pressure to reduce flock prevalence. A major emphasis was placed on heightened strict biosecurity rules on broiler farms, thorough cleaning and disinfection of houses between flocks, and pest control. Rigorous multi-step cleaning and disinfection of the live haul crates and trucks was also initiated. These initiatives began early in 2000. Freezing of products from all flock lots found positive on pre-slaughter sampling, and the price penalty to the producer for positive flock lots, ensured continued producer motivation to maintain high standards. This may have reduced the otherwise expected importance of an all-in-all-out system.

Fifteen percent of the farms in our study were excluded from the analysis due to missing data for one or more variables. In order to assess what effect this might have had on our results, we re-analysed the data using all 33 farms, excluding the four variables with missing data (an all-in-all-out policy, manure spreading in the summer season, manure spreading in the winter season, and manure storing). We found that, whether we used 33 farms or 28 farms in our models, our estimates for other domestic livestock on the farm, farm water source, and median flock size were consistent. However, when we used 33 farms, the presence of other commercial poultry and the number of houses did not remain in any of the backward elimination models. It was evident that there was confounding between the number of houses, the presence of other poultry, and manure spreading & storing practices on the farm. Therefore, by including manure management practices (and hence analysing data from fewer farms), we likely have better estimates for these potentially important risk factors for flock colonization at the farm level, and the impact of other variables appears stable.

## Conclusion

Our study has shown that, regardless of the modelling approach, there are a number of farm-level factors that appear to be important predictors for the risk of *Campylobacter *on broiler farms in Iceland, providing a basis for farm-level interventions. Median flock size was a consistent predictor in all of the models. Although it may be a surrogate for factors that increase the likelihood of exposure to *Campylobacter*, such as water, air or personnel, limiting flock sizes is an intervention that requires investigation. Farm water source was included in most of the models, suggesting the possible role of water in the transmission of *Campylobacter*. The use of official water if possible, and potentially, treating non-official water sources, may assist in reducing colonization. In addition, studies that explore more indirect factors, such as the type of well, the method of water treatment, and animal density in the region are warranted. Manure management practices revealed some interesting results. Storing manure in piles on the farm property had a protective effect, whereas spreading manure led to an increased proportion of colonized flocks. Since little is known about the causal effects of these practices, studies that evaluate the survival of *Campylobacter *in spent litter under various environmental conditions should be carried out before recommendations can be made. Nonetheless, farms experiencing a high prevalence of *Campylobacter *may wish to discontinue spreading manure on fields and pasture. Increasing numbers of broiler houses on the farm may increase risk through difficulties in maintaining strict hygiene or biosecurity practices, therefore, for new broiler farms, consideration should be given to limiting the number of houses built. The protective effect of livestock and the conflicting results for other poultry on the farm were unexpected and inconsistent with other studies. For the latter, further refinement in the classification of other poultry types may be necessary in order to properly assess their impact on broiler flock colonization.

## Competing interests

The authors declare that they have no competing interests.

## Authors' contributions

MTG performed the statistical analysis and drafted the manuscript. WM, OB and SAM critically evaluated the analysis and revised the manuscript for intellectual content. JR was involved in the conception, design and coordination of the study, data collection and data quality checks, and revision of the manuscript for intellectual content. JRB was involved in the conception and design of the study, the design of the epidemiological database structure, building the data query for the broiler farm analysis, and revision of the manuscript for intellectual content. RL was involved in the conception, design and coordination of the study, data management and final data quality control, and revision of the manuscript for intellectual content. All authors read and approved the final manuscript.
